# Supplementary material for: Hepatitis E seroprevalence in a German cohort of patients with inflammatory bowel diseases
Source: PLoS One. 2020 Oct 7;15(10):e0239825. doi: 10.1371/journal.pone.0239825 (PMC7540852; doi:10.1371/journal.pone.0239825)
Supplement: Attachment — Submitted filename: Response to Reviewers.docx [file pone.0239825.s002.docx]

**PLOS ONE em@editorialmanager.com** [**über**](https://support.google.com/mail/answer/1311182?hl=de) **med.uni-heidelberg.de**

31.07.2020, 16:32 (vor 9 Tagen)

an Peter

Nachricht übersetzen

Deaktivieren für: Englisch

PONE-D-20-21089
Hepatitis E seroprevalence in a German cohort of patients with Inflammatory Bowel Diseases
PLOS ONE

Dear Dr. Hoffmann,

Thank you for submitting your manuscript to PLOS ONE. After careful consideration, we feel that it has merit but does not fully meet PLOS ONE’s publication criteria as it currently stands. Therefore, we invite you to submit a revised version of the manuscript that addresses the points raised during the review process.

==============================
Your manuscript was reviewed by 2 experts in the field. Both identified many important problems in your submission which require your careful attention. Please review the attached comments and provide point-by-point responses. 
==============================

Please submit your revised manuscript by Sep 14 2020 11:59PM. If you will need more time than this to complete your revisions, please reply to this message or contact the journal office at [plosone@plos.org](mailto:plosone@plos.org). When you're ready to submit your revision, log on to <https://www.editorialmanager.com/pone/> and select the 'Submissions Needing Revision' folder to locate your manuscript file.

Please include the following items when submitting your revised manuscript:

- A rebuttal letter that responds to each point raised by the academic editor and reviewer(s). You should upload this letter as a separate file labeled 'Response to Reviewers'.
- A marked-up copy of your manuscript that highlights changes made to the original version. You should upload this as a separate file labeled 'Revised Manuscript with Track Changes'.
- An unmarked version of your revised paper without tracked changes. You should upload this as a separate file labeled 'Manuscript'.

If you would like to make changes to your financial disclosure, please include your updated statement in your cover letter. Guidelines for resubmitting your figure files are available below the reviewer comments at the end of this letter.

If applicable, we recommend that you deposit your laboratory protocols in [protocols.io](http://protocols.io) to enhance the reproducibility of your results. Protocols.io assigns your protocol its own identifier (DOI) so that it can be cited independently in the future. For instructions see: <http://journals.plos.org/plosone/s/submission-guidelines#loc-laboratory-protocols>

We look forward to receiving your revised manuscript.

Kind regards,

Yury E Khudyakov, PhD
Academic Editor
PLOS ONE

Journal Requirements:

When submitting your revision, we need you to address these additional requirements.

1. Please ensure that your manuscript meets PLOS ONE's style requirements, including those for file naming. The PLOS ONE style templates can be found at

<https://journals.plos.org/plosone/s/file?id=wjVg/PLOSOne_formatting_sample_main_body.pdf> and

<https://journals.plos.org/plosone/s/file?id=ba62/PLOSOne_formatting_sample_title_authors_affiliations.pdf>

 The changes were made. Now the manuscript meets PLOS ONE’s style requirements, including those for file naming.

2. Please provide the full name of the ethics committee which approved this study in the methods section of your manuscript.

 The full name of the ethics committee was provided in Methods, page 5.

3.Thank you for stating the following in the Competing Interests section:

[Annika Gauss received travel fees from Abbvie and Janssen, speaker’s fees from Janssen, MSD, Tillotts, and Takeda, and consultation fees from Janssen and AMGEN; Peter Hoffmann received travel fees from Abbvie and Janssen and speaker’s fees from Janssen; which might lead to a conflict of interest. All other authors have no conflicts of interest to declare.].

Please confirm that this does not alter your adherence to all PLOS ONE policies on sharing data and materials, by including the following statement: "This does not alter our adherence to  PLOS ONE policies on sharing data and materials.” (as detailed online in our guide for authors http://journals.plos.org/plosone/s/competing-interests).  If there are restrictions on sharing of data and/or materials, please state these. Please note that we cannot proceed with consideration of your article until this information has been declared.

 The confirmation was done, p1.

Please include your updated Competing Interests statement in your cover letter; we will change the online submission form on your behalf.

 The updated Competing Interests statement is now included in the cover letter.

Please know it is PLOS ONE policy for corresponding authors to declare, on behalf of all authors, all potential competing interests for the purposes of transparency. PLOS defines a competing interest as anything that interferes with, or could reasonably be perceived as interfering with, the full and objective presentation, peer review, editorial decision-making, or publication of research or non-research articles submitted to one of the journals. Competing interests can be financial or non-financial, professional, or personal. Competing interests can arise in relationship to an organization or another person. Please follow this link to our website for more details on competing interests: http://journals.plos.org/plosone/s/competing-interests

[Note: HTML markup is below. Please do not edit.]

Reviewers' comments:

Reviewer's Responses to Questions

**Comments to the Author**

1. Is the manuscript technically sound, and do the data support the conclusions?

The manuscript must describe a technically sound piece of scientific research with data that supports the conclusions. Experiments must have been conducted rigorously, with appropriate controls, replication, and sample sizes. The conclusions must be drawn appropriately based on the data presented.

Reviewer #1: Partly

Reviewer #2: Yes

2. Has the statistical analysis been performed appropriately and rigorously?

Reviewer #1: No

Reviewer #2: Yes

3. Have the authors made all data underlying the findings in their manuscript fully available?

The [PLOS Data policy](http://www.plosone.org/static/policies.action#sharing) requires authors to make all data underlying the findings described in their manuscript fully available without restriction, with rare exception (please refer to the Data Availability Statement in the manuscript PDF file). The data should be provided as part of the manuscript or its supporting information, or deposited to a public repository. For example, in addition to summary statistics, the data points behind means, medians and variance measures should be available. If there are restrictions on publicly sharing data—e.g. participant privacy or use of data from a third party—those must be specified.

Reviewer #1: Yes

Reviewer #2: Yes

4. Is the manuscript presented in an intelligible fashion and written in standard English?

PLOS ONE does not copyedit accepted manuscripts, so the language in submitted articles must be clear, correct, and unambiguous. Any typographical or grammatical errors should be corrected at revision, so please note any specific errors here.

Reviewer #1: Yes

Reviewer #2: Yes

5. Review Comments to the Author

Please use the space provided to explain your answers to the questions above. You may also include additional comments for the author, including concerns about dual publication, research ethics, or publication ethics. (Please upload your review as an attachment if it exceeds 20,000 characters)

Reviewer #1: This study investigates the anti HEV seroprevalence in patients with infallamtory bowel disease. The anti HEV seroprevalence in patients with M. Crohn was lower in comparison to patients with Colitis ulcerosa. I calculated the p-value for this finding (Chi Square) and this gave 0.05, borderline significance....

The result of the statistical comparison has now been included in the discussion section on page 14. Due to the fact that the difference between HEV seroprevalences in patients with Crohn’s disease and those with ulcerative colitis was only borderline , we decided to not overemphasize it. Thus, the statistical comparison was not included in the abstract.

A comparison to previously published cohorts of the German general population or healthy blood donors is difficult. The Mikrogen tests have been strongly improved in the last 5 years and currently these assays have a high sensitivity and specifity..... but 10 years ago these serological tests had some problems..... thus you cannot compare all published data easily....
Furthermore there are geographical variations within Germany....
if you want to compare your data with healthy people, you need your own cohort and not previously published data from various areas, using various assays....

Thank you for this important comment. Unfortunately we currently do not have our own healthy cohort to compare as a control group.

The seroprevalence was associated with anti-interleukin-treatment..... Patients getting this treatment are usually suffering from a more severe course, thus it is more likely they get more frequently blood products or other medical interventions harboring the risk of HEV transmission.

This important consideration has now been included in the discussion section on page 14.

General testing of blood donations for HEV has been started in Germany in January 2020.

Thank you very much for this comment. The fact has been corrected in the introduction, page 4.


If you want to detect chronic HEV infection you need to test by PCR.... I suggest to do this to improve the project....

Thank you for this advise. We tested HEV PCR to detect chronic HEV infection (page 6)

Reviewer #2: This is a well written manuscript which describes a straightforward study on the possible relation between HEV seroprevalence and inflammatory bowel disease (IBD) in two groups of patients, those with Crohn’s disease (CD) and those with ulcerative colitis (UC).
The data are relatively new. The retrospective, non-controlled format is a limitation, which is rightfully mentioned by the authors.
Some points need to be considered and adjusted before this paper is suitable for publication in PlosOne.

General comments:
1. The main risk factor associated with HEV seroprevalence is (older) age. This has been presented in many studies and is understandable considering the fact that if a person seroconverts, this is for life. So there is an accumulation of HEV seropositives in older age cohorts, also in these IBD patients. The other described risk factor in this study is the duration of anti-interleukin 12/23 treatment. This is described in the last part of Table 2, with a median duration of 0 days for both anti-HEV IgG negatives and anti-HEV IgG positives, but the ranges of duration differ. This factor is only found in the CD patients but not for the UC patients (Table 4). Actually, the multivariate analysis in Table 3 shows a 95%CI starting with 1.000. So this finding is at best a very weak relation. This finding is elaborated on in a paragraph in the Discussion. I think it should be added there that the association is very weak.

The weakness of the association has now been emphasized in the discussion, page 15.

2. As mentioned by the author, another limitation is that no data are present on food intake. Especially since this is an IBD study those data would have been interesting. Both in the introduction and discussion more references can be incorporated on the relation between food intake-following a specific dietary (vegetarian) lifestyle- and HEV serostatus. See for example references such as:
- Slot et al, Meat consumption is a major risk factor for hepatitis E virus infection. PLoS One. 2017;12.
-Alberts et al, Hepatitis E virus seroprevalence and determinants in various study populations in the Netherlands, PlosOne 2018
-Wong et al, The study of seroprevalence of hepatitis E virus and an investigation into the lifestyle behaviours of the aborigines in Malaysia. Zoonoses Public health, 2020.

The above-mentioned references have been inserted in the introduction or discussion.

3. Another factor which might be of influence on the detected seroprevalence is the HEV IgG test used. In this study it is from the company Mikrogen. Please look at studies that discuss the choice of the HEV serology tests (for example Pas et al, J. Clin Virol 2013; Li et al, Liver Int 2020) and add a sentence on this in the Discussion.

This problem is now addressed in the discussion section, page 14.

Specific comments
Abstract
-In Methods, the second sentence starts with a number which is not very comprehensive. Consider rephrasing this sentence: Of patients visiting the IBD outpatient clinic….
-Consider also mentioning in the background what the HEV seroprevalence (or the range) is for a ‘general population’ in Germany in the abstract.

The changes have been made, as suggested, page 2.

Introduction
In the first paragraph especially blood donor populations are mentioned. To compare German data to those of other countries also Dutch data may be relevant. Please consider incorporating some of the following studies:
-Hogema et al. Transfusion 2016; Sadik et al. BMC-ID 2016; Mooij et al. BMC-ID 2018

Two of the suggested references have been added added in the introduction.

Results
-In the text ‘mean’ is mentioned, for example in the second paragraph: ‘mean age’ and ‘mean disease duration’, whereas this is ‘median’ in Table 1. Median, as is also mentioned in Methods, is to be preferred. Please make adjustments everywhere in the text where applicable.
- Second paragraph, second line: please change into ‘biological therapy and 65.5% had'.
- Tables 2 and 4: Please adjust ‘Age at diagnosis’

Changes were made as advised.

Discussion
Fourth paragraph, please consider changing the text into: ‘a greater susceptibility to HEV and higher infection rate’.

Changes were made as advised.

6. PLOS authors have the option to publish the peer review history of their article ([what does this mean?](https://journals.plos.org/plosone/s/editorial-and-peer-review-process#loc-peer-review-history)). If published, this will include your full peer review and any attached files.


If you choose “no”, your identity will remain anonymous but your review may still be made public.


**Do you want your identity to be public for this peer review?** For information about this choice, including consent withdrawal, please see our [Privacy Policy](https://www.plos.org/privacy-policy).

Reviewer #1: No

Reviewer #2: **Yes:** Sylvia Bruisten

[NOTE: If reviewer comments were submitted as an attachment file, they will be attached to this email and accessible via the submission site. Please log into your account, locate the manuscript record, and check for the action link "View Attachments". If this link does not appear, there are no attachment files.]

While revising your submission, please upload your figure files to the Preflight Analysis and Conversion Engine (PACE) digital diagnostic tool, <https://pacev2.apexcovantage.com/>. PACE helps ensure that figures meet PLOS requirements. To use PACE, you must first register as a user. Registration is free. Then, login and navigate to the UPLOAD tab, where you will find detailed instructions on how to use the tool. If you encounter any issues or have any questions when using PACE, please email PLOS at [figures@plos.org](mailto:figures@plos.org). Please note that Supporting Information files do not need this step.
